# Cross-Sectional Analysis of Fall-Related Factors with a Focus on Fall Prevention Self-Efficacy and Self-Cognition of Physical Performance among Community-Dwelling Older Adults

**DOI:** 10.3390/geriatrics8010013

**Published:** 2023-01-14

**Authors:** Shintaro Hayashi, Yuka Misu, Toshimasa Sakamoto, Taisei Yamamoto

**Affiliations:** 1 Graduate School of Rehabilitation, Kobe Gakuin University, Kobe 651-2180, Japan; 2 Department of Physical Therapy, Faculty of Rehabilitation, Morinomiya University of Medical Sciences, Osaka 559-8611, Japan; 3 Department of Physical Therapy, Faculty of Rehabilitation, Kobe Gakuin University, Kobe 651-2180, Japan; 4 Department of Physical Therapy, School of Health Sciences, Tokyo International University, Kawagoe 350-1197, Japan

**Keywords:** fall prevention, fall prevention self-efficacy, self-cognition, physical performance, cognition error, overestimation, timed cognition

## Abstract

This study aimed to determine how fall prevention self-efficacy and degree of deviation in self-cognition of physical performance, which have recently received attention for their potential to explain falls in combination with a wide variety of fall-related factors, as well as affect falls. Older adults using day-care services (*n* = 27 with six men, mean age: 81.41 ± 7.43 years) were included in this study. Fall history in the past year, the modified fall efficacy scale (MFES), and physical performance and cognition errors were examined by evaluating the functional reach test (FRT), the stepping over test, and the timed up and go test (TUG), along with a questionnaire. In the fall (*n* = 14) and non-fall (*n* = 13) groups, logistic regression analysis using Bayesian statistical methods was used to identify factors associated with falls. The odds ratios for the MFES ranged from 0.97 to 1.0, while those of cognition-error items ranged from 3.1 to 170.72. These findings suggested that deviation in self-cognition of physical performance, particularly overestimation of timed cognitive ability, was a factor with more explanatory power for fall history. Future studies should analyze differences by disease and age group, which were not clarified in this study, to identify more detailed fall risk factors.

## 1. Introduction

In Japan, where the super-aging society continues to accelerate, advances in medical technology tend to expedite the early discharge of hospitalized patients, while the social security system faces various social challenges, such as reductions in medical costs and an increase in the number of households with aged caregivers and individuals living alone [1]. In order to solve these issues, each citizen is required to improve their interest in health promotion and disease prevention, implement self-management of their health, and devise methods to continue living at home [2]. In this regard, the 2019 vital statistics [3] reported that, among the 39,184 deaths with “unintentional causes of death”, 23.4% resulted from falls. Furthermore, even if falls do not result in death, traumatic injuries from falls are associated with hospitalization and treatment and have a high likelihood of decreasing physical function and activity, leading to a bedridden status [4]. Falls also cause a decline in quality of life (QOL), which also includes psychological aspects [4]. Therefore, fall prevention is an important issue in the rehabilitation of older adults.

Since the 1980s, numerous studies on fall prevention have been reported in Japan and abroad [5,6], and fall prevention measures have been proposed from various perspectives, including exercise programs. However, the fall rate among older adults has not improved worldwide. In the United States, approximately 28.7% of older adults aged 65 years and older fell in 2014 [7]. Additionally, Hartholt et al. [7] revealed that the age-adjusted fall-related mortality rate among individuals aged 75 and older increased more than two folds, from 51.6 per 100,000 individuals in 2000 to 122.2 per 100,000 individuals in 2016. In Japan, falls are increasingly present in surveys of the leading causes of the need for long-term care, with “fractures and falls” generally ranking third to fifth in 2001–2019. This category accounted for approximately 10% of the total causes of the need for long-term care, which increased to 12.5% in 2019 [8].

There are various reports of factors contributing to falls among older adults, including those by Tinetti et al. [9,10]. A wide range of fall-related factors have been established, including internal factors related to the subject’s physical functional aspects and external factors, such as the surrounding environment. Furthermore, it has become clear that both physical risk and behavioral factors of the subject are risk factors that interact with internal and external factors to cause falls [9,11]. Therefore, when considering the appropriate fall prevention measures for individual subjects, single measures focusing on specific factors were reportedly insufficient [12]. On the other hand, multidimensional interventions, such as conducting comprehensive fall risk assessments and incorporating multiple types of exercise programs and educational guidance, are considered more effective in fall prevention [12,13].

In recent years, the degree of self-efficacy in the behavior of a person and the degree of deviation from reality in a person’s cognition of their physical performance have also been explored as new risk factors for falls [14,15,16,17,18,19]. The occurrence of falls is considered to be influenced by a person’s ability to respond and act appropriately in an environment where there is a fall risk, where internal and external factors interact [9,11]. Therefore, when considering fall prevention, self-efficacy, a subjective evaluation of the degree of adaptation to fall risk, and appropriate cognition of physical performance, in particular, are not single explanatory variables as has been suggested, but rather complex factors that can explain falls in a multifaceted manner, including internal and external factors. Therefore, these two fall risk factors are considered to be of high importance among all fall risk factors.

In previous studies performed, in the 1990s, Tinetti et al. defined the fear of falling (FOF) as a psychobehavioral dimension of fear of falling that may be a fall risk factor [19]. Subsequently, the fall efficacy scale (FES) was developed and widely used to score and assess fear of falling [14,19]. The FES is designed to evaluate the degree of confidence in one’s ability to perform activities of daily living (ADL) without falling, with higher scores indicating greater confidence in one’s ability not to fall and higher self-efficacy in fall prevention, and lower scores indicate a greater fear of falling. Fall prevention studies have reported the importance of interventions to reduce FOF, i.e., to increase fall prevention self-efficacy [9,14]. However, falls have also been reported to occur when the level of fall prevention self-efficacy was excessively low or high [15,20]. In other words, it has been pointed out that older adults who are overconfident that they can move without falling may also be at fall risk. Additionally, deviations in older adults’ self-cognition of their physical performance were reportedly associated with falls [16,17,18,21,22], with both underestimation and overestimation of self- cognition as risk factors for falls [17,23,24]. Some reports have suggested that the association between the deviation in self-cognition and the occurrence of falls may be due to the inability to properly visualize the movement due to the reduced function of the simulation mechanism in the brain [25,26]. The fall group was more likely to have discrepancies between self-cognition and actual results regarding the height of the stepping over test and the time required for the gait test [26,27]. It is noteworthy, however, that these previous studies differ in their views on fall prevention self-efficacy and deviations in self-cognitions, which are considered to be fall risk factors. In addition, review reports of studies of fall prevention interventions focusing on these factors suggest that exercise interventions can improve low self-efficacy, but they also point out that results vary from study to study and that the evidence is insufficient [28]. There are also no reports of intervention studies that have attempted to improve deviations in self-cognitions, and the effectiveness of these interventions was not completely clear.

Therefore, this study aimed to clarify how the degree of fall prevention self-efficacy and the degree of deviation in self-cognition of physical performance, which are considered to be of high importance among fall-related factors, influence falls.

## 2. Materials and Methods

### 2.1. Participants

**Figure 1:**
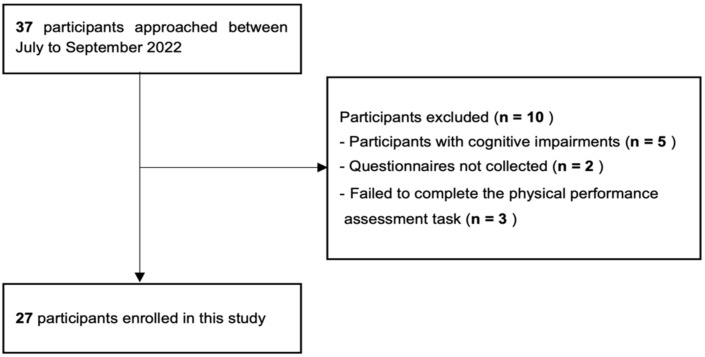
Flow chart of the participant selection process.

Community-dwelling older adults who use day care services provided by long-term care insurance were invited to participate in this study from July to September 2022. Of these, 37 older adults who gave written consent to participate were included in the study. The selection criteria were as follows: individuals aged >65 years, who were able to walk indoors (including those requiring the use of walking aids). The exclusion criteria were as follows: individuals with a marked decline in cognitive function with a score < 21 on the Revised Hasegawa Dementia Scale (HDS-R) or <24 on the Mini-Mental State Examination (MMSE), those with a marked change in walking ability in the past 6 months, those with difficulty answering the self-administered questionnaire, and those with difficulty completing the physical function and motor ability assessment tasks. Ten participants met the exclusion criteria, and the total number of individuals finally included in the analysis was 27 (6 men and 21 women, mean age: 81.41 ± 7.43 years) (Figure 1).

### 2.2. Study Design

The cross-sectional study was conducted with the following questionnaire survey and assessment of physical performance and self-cognition. This study complied with the guidelines of the Declaration of Helsinki and was approved by the Ethics Committee of Kobe Gakuin University (Institutional Review Board: 21-09). All participants provided informed consent.

### 2.3. Instruments

#### 2.3.1. Questionnaire Survey

The questionnaire consisted of the following four items:

1. History of falls in the past year and conditions at the time of the fall.
2. Learning experience about fall prevention (e.g., attending courses, receiving individual guidance from therapists, and collecting information from pamphlets, TV, and the internet).
3. Fall prevention self-efficacy (modified fall efficacy scale [MFES]) [29]

The MFES was modified from the fall efficacy scale (FES) [19] developed by Tinetti et al. The MFES consists of 14 items to be subjectively evaluated on a scale of 0 to 10 in terms of confidence in the ability of a person to perform daily indoor and outdoor activities without falling.

4. Life-Space Assessment (LSA) [30,31]

This scale examines the extent of living space in five areas, including “bedroom”, “outside bedroom”, “yard or neighborhood”, “further away than neighborhood”, and “out of town”, in four weeks.

#### 2.3.2. Assessment of Physical Performance and Self-Cognition

In this study, three types of assessments of physical performance and self-cognition were conducted, including the functional reach test (FRT), the stepping over test (to evaluate stepping over obstacles), and the timed up and go test (TUG), all of which differ in terms of the spatial and timed cognition.

In all of the physical performance assessments, the examiner gave demonstrations so that the subjects could fully understand the testing procedures in advance. In the testing procedure, the participant’s estimated values were recorded without practicing the movement of physical performance. Subsequently, actual measurements were taken, without revealing the participant’s estimated values, and were recorded as the results.

1. FRT: measurement of the maximum reach distance of the upper limb forward from a stationary standing position (spatial cognition).

The FRT method was adopted with reference to previous studies, with modifications to the original method of Duncan et al. [16,18,32]. The reference limb position was the shoulder joint in 90° flexion, elbow joint in extension, forearm in pronation, and fingers in extension, where the reaching motion was performed in the static standing position, and the distance to the point reached by the tip of the third finger was defined as the reach distance. The reach distance was recorded by the examiner, who marked the position of the tip of the third finger at the shoulder joint position.

The estimated value of the maximum reach distance was evaluated based on the original method of Okada et al. [18]. First, the participant was asked to check the fingertip position in the FRT reference limb position and to lower the upper limb. Next, the examiner held a ruler at the height of the participant’s acromion and gradually approached the measuring board from the participant’s distal end. The participant was instructed to say “stop” at what they visually judged to be their maximum estimated point of reach. Subsequently, minor positional corrections were allowed by verbal instruction. The examiner recorded this distance as the estimated value of the FRT, and then asked the participant to perform the FRT without changing the height of the upper limb, and the maximum reach distance was measured as the actual value.

2. Stepping over test: measurement of the height at which a bar was stepped over by lifting one leg on each side (spatial cognition)

The stepping over test was used to evaluate stepping over obstacles with reference to previous studies [17,21]. The participant was asked to observe the stepping over motion at a 2-m distance from the stepping over measurement device, and a demonstration was included. The participant stepped over the bar from the front, one foot at a time, without support of the upper limb and in any order with the lower limbs. Both lower limbs completed the stepping over motion.

In the evaluation of the estimated value, the examiner moved the bar slowly from the bottom to the top or in the reverse order, and the participant was asked to answer “stop” at the point where they judged that the maximum height that they could step over had been reached. In this case, minor positional corrections by verbal instruction were allowed. The bar was raised or lowered twice each, for a total of four times, and the average of the four trials was used as the estimated value.

In the evaluation of the actual value, the participant fixed the bar at the average height calculated in the evaluation of the estimated values and actually stepped over the bar. Failure was considered if the participant touched the bar in the middle of the test. The bar was then lowered by 3 cm, and the test was conducted again. Success was considered if the participant was able to step over the bar twice in a row by moving back and forth. The bar was then heightened by 3 cm, and the participant stepped over the bar again. This procedure was repeated, and the maximum height at which the participant finally succeeded was recorded as the actual measured value.

Since the degree of ability to step over obstacles depends on the lower limb length [33], the estimated and actual values were divided by the lower limb length, and the result of the stepping over motion was used as its score. In this study, the adjusted lower limb length was defined as the height from the floor to the greater trochanter.

3. TUG: measurement of the time required for standing up out of a chair, walking 3 m, turning around, walking back to the chair, and sitting down (timed cognition)

TUG was performed according to the procedure used in previous studies, based on the original method of Diane et al. [34]. The time to stand up from the chair, walk around a landmark (pole) 3 m away, and sit in the chair again was measured. Walking was evaluated under two conditions, once at normal walking speed and once at maximum rapid walking speed.

In the evaluation of the estimated value, the participant was asked to imagine the movement. At the same time, the participant operated the stopwatch by himself and recorded the estimated values. For the demonstration during measurement, the participant was asked to “walk as fast as you usually walk” for the first time and “walk as fast as you can” for the second time. The direction, in which the participant turned around the pole (a landmark), was left free.

In the evaluation of the actual value, the examiner operated a stopwatch and recorded the actual time required to complete the performance.

### 2.4. Data Collection

The collection of information and survey of research participants were conducted with the cooperation of physical therapists and occupational therapists at collaborating facilities. In order to protect personal information, all data of the study participants were managed using ID numbers and handled after anonymization as information from which individuals could not be identified.

### 2.5. Deviation of Self-Cognition of Physical Performance

The degree of deviation in self-cognition of physical performance can be indicated by defining the difference or ratio between estimated and actual measured values as “cognition error” [16,17,21,22]. In this study, since cognition error is considered a fall-related factor, it is necessary to distinguish between overestimation and underestimation of physical performance. Furthermore, standardizing the results of different assessments of physical performance is desirable to facilitate comparison. Therefore, the deviation from self-cognition of physical performance was calculated by dividing the estimated value obtained from each assessment by the actual value, which was used as the “cognition error” value.

For the FRT and stepping over test, a cognition error > 1 was considered an overestimation, and a value < 1 was considered an underestimation. For TUG, a cognition error > 1 was considered an underestimation, and a value < 1 was considered an underestimation.

### 2.6. Statistical Analysis

To explore factors associated with falls, the presence or absence of a fall history was used as the objective variable. Correlation analysis was then conducted with the objective variable for each factor, which could be considered as associated with falls and could serve as an explanatory variable. Logistic regression analysis was conducted using the variables, for which significant differences were detected, as explanatory variables.

Odds ratios (OR) were calculated and compared among the variables to explore differences in the degree, to which each factor was associated with fall occurrence.

In this study, since the parameters of each variable in logistic regression analysis were considered to be dependent on probability distributions, Bayesian statistics was used as the statistical method [35,36]. Bayesian statistics is a method that allows the parameters and overall trend to be estimated as random variables using random sampling, even with a small number of samples, in contrast to conventional statistical methods that require a large number of samples to be collected and verified [35,36]. In this study, random numbers were generated by the Markov chain Monte Carlo methods (MCMC) using the Hamiltonian Monte Carlo method, and the posterior distribution of the parameters for each fall-related variable was estimated. Statistical analysis was conducted with the software R (ver. 4.1.1, R Foundation for Statistical Computing, Vienna, Austria) and rstan package (ver. 2.21.2, NumFOCUS, Austin, TX, USA).

In the stan package, the Bernoulli distribution was assumed with the presence of a history of falls as the binomial objective variable, and four chains were generated with an iteration of 2000 (chains = 4) for random number generation, each with 1000 warm-up periods (warm up = 1000). For data recruitment, thinning was set as 1. A total of 4000 random numbers obtained from these conditions were used to approximate the posterior distribution of each parameter. In all estimations, the Gelman-Rubin statistic (R hat [Rˆ]), a measure of convergence, was considered appropriate if it was <1.1.

## 3. Results

### 3.1. Participants

Twenty-seven participants were divided into two groups according to the presence or absence of falls in the past year. Fourteen (51.9%) participants were in the fall group (four men and 10 women, mean age: 79.64 ± 8.22 years), and 13 (48.1%) were in the non-fall group (two men and 11 women, mean age: 83.31 ± 6.60 years). 

Twelve (85.7%) participants of the fall group and eight (61.5%) of the non-fall group indicated that they had previously learned about fall prevention (Table 1).

### 3.2. Results of the Questionnaire and of the Physical Performance and Self-Cognition Error

The results of the questionnaire and each physical performance assessment in fall and non-fall groups were shown in Table 2.

For the two groups, with fall history as the objective variable and other factors as explanatory variables, correlations were first determined for each factor in order to explore their inter-relationships. The test for each correlation with fall history was selected after consideration of the differences in each measure (Table 3).

In the logistic regression analysis to determine the association between fall history and each factor, variables with a significant correlation with the presence of falls were entered as explanatory variables. The selected variables were age, MFES, LSA, and grip.

Next, logistic regression analysis was conducted with one variable for each of the four cognition errors in the FRT, the stepping over test, and TUG (at normal speed and maximum rapid speed). The regression coefficients were 1.13 (OR: 3.10, 95% confidence interval [CI]: −0.35 to 3.05, Rhat = 1) for the FRT, 2.25 (OR: 9.49, 95% CI: −0.01 to 5.63, Rhat = 1) for the stepping over test, 5.14 (OR: 170.72, 95% CI: −1.23 to 13.07, Rhat = 1) for TUG (normal speed), and 5.13 (OR: 169.02, 95% CI: −1.23 to 12.62, Rhat = 1) for TUG (rapid speed).

On the other hand, the regression coefficient for fall prevention self-efficacy was −0.01 (OR: 0.99, 95% CI: −0.05 to 0.03, Rhat = 1) for the FRT, 0.0 (OR: 1.0, 95% CI: −0.04 to 0.06, Rhat = 1) for the stepping over test, −0.03 (OR: 0.97, 95% CI: −0.08 to 0.02, Rhat = 1) for TUG (normal speed), and −0.02 (OR: 0.98, 95% CI: −0.07 to 0.02, Rhat = 1) for TUG (rapid speed) (Table 4, (a)–(d)). Regarding the estimates calculated for each variable, the convergence criterion Rˆ was <1.1 for all variables.

**Figure 2:**
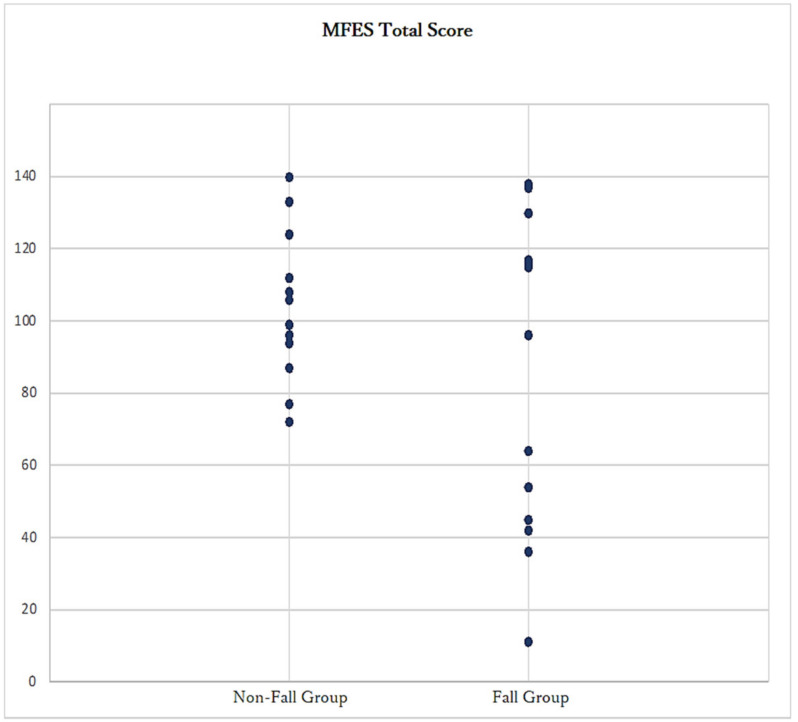
Distribution of the modified fall efficacy scale (MFES) scores for the fall and non-fall groups.

The mean score of fall prevention self-efficacy was 83.21 ± 42.67 for the fall group and 103.23 ± 20.30 for the non-fall group. While there was no significant difference between the two groups, the correlation coefficient was −0.294 for the correlation with the presence of falls (Table 2 and Table 3). However, regarding the score distribution, no respondents in the fall group had scores within the vicinity of the mean, indicating a polarization between distributional tendencies toward low and high scores (Figure 2).

The mean values of TUG at normal speed and maximum rapid speed, both of which had high ORs for fall occurrence in each cognition-error item, were not significantly different between two groups, and most cognition error values were <1 for the fall group among all participants (Table 2).

## 4. Discussion

### 4.1. Results and Characteristics of the Study Participants

In this study, 14 (51.9%) of the 27 participants included in the analysis had a fall history in the past year. The fall rate of these participants was higher than those reported in Japan and abroad for community-dwelling older adults (approximately 20–30% or higher with one or more falls in a year) [6]. This discrepancy might be related to the fact that our study participants were limited to those who used day-care services and had some type of disability. Analysis of factors associated with the presence or absence of falls revealed that age, fall prevention self-efficacy, LSA, and grip strength were significant factors. This finding suggested that lower scores on fall prevention self-efficacy and LSA were associated with the occurrence of falls, as shown in previous studies. However, in contrast to previous findings, lower age and higher grip strength were associated with the occurrence of falls [9,10]. This discrepancy might have also been influenced by the fact that this study was conducted on disabled rather than healthy older adults, and that the fall group included individuals with a high degree of nursing care needs, regardless of age or grip strength factors that could be explained as the effects of aging-related changes.

Regarding the results of self-cognition errors in the assessment of physical performance related to balance ability, errors in all tasks (FRT, stepping over test, TUG with normal speed and maximum rapid speed) were correlated with the occurrence of falls (Table 3). Furthermore, regarding the cognition error values, all tasks overestimated physical performance (Table 2).

Risk factors for falls were reportedly related to decreased physical performance and balance ability [9,10]. However, falls are not only caused by a decline in physical performance, but also by a failure to appropriately perceive and respond to fall factors in situations where a falling risk exists. The results of this study also suggested that greater self-cognition errors were more strongly associated with falls.

### 4.2. Association between Self-Cognition Errors in Physical Performance and Falls

Comparing the results of the cognition errors in the four physical performance assessments, ORs for the occurrence of falls were 170.72 for TUG (normal speed) and 169.02 for TUG (rapid speed), which clearly indicated that these cognition errors were predictors of a significantly higher risk of falling. This measurement and the comparison of the estimated and actual times to perform a movement in timed cognition were also used as a method to measure motor imagery ability as mental chronometry [37,38]. In mental chronometry, when timed cognition errors were large, motor imagery ability was low [38]. In a study by Nakano et al. [22], a decrease in motor imagery ability was associated with a higher incidence of falls in older adults using long-term care insurance services. Haruyama et al. [27] also studied community-dwelling stroke patients after discharge from a rehabilitation hospital using TUG and reported that overestimation of TUG speed was a significant predictor of falls six months after discharge. In this study, the cognition error values for the fall group were 0.6 ± 0.21 for TUG at normal speed and 0.55 ± 0.27 for TUG at maximum rapid speed (Table 2), both of which indicated that the timed cognition errors were large and overestimated. This finding suggested that the motor imagery ability might be decreased in the group that had experienced falls and was more closely related to the occurrence of falls.

Motor imagery ability is controlled by the activity of the supplementary motor cortex, prefrontal cortex, particularly the orbitofrontal cortex, as a simulation mechanism in the brain [25,26]. In a study by Sakurai et al. [39], overestimators in the stepping over test showed reduced brain metabolism in the orbitofrontal cortex, and a subsequent follow-up study showed that they had a higher rate of falls. Another report indicated that older adults with low fall prevention self-efficacy had reduced brain metabolism in the supplementary motor cortex [39]. These findings collectively suggested that, among the multiple risk factors for falls in this study, the inability to properly perform motor imagery might be more associated with the occurrence of falls due to functional impairment of simulation mechanisms in the brain.

The subjects in this study tended to overestimate their spatial cognitive abilities, regardless of whether they had fallen or not (Table 2). In particular, with regard to cognition errors in physical performance, previous studies have suggested that large cognition errors are a fall risk factor, but the breakdown of these errors included both underestimation and overestimation [16,17,18]. In the present study, however, the majority of subjects overestimated the risk, and the odds ratio revealed that this factor has high explanatory power as a fall risk factor. This difference may have resulted from differences in the demographics of the subjects, as previous studies with younger or healthy older adults included many underestimators, whereas this study included older adults who were already living their daily lives with some disability. Therefore, it can be inferred that the participants in this study included some who were unable to recall their current accurate performance due to their disability or who did not have an updated image of their performance when they were more mobile before their disability when making predictions for each performance [22,27]. Furthermore, both the fall and non-fall groups in this study may be at similar risk, and even if they have not fallen, their spatial cognition and motor imagery abilities are impaired, and these impairments may represent a future fall risk.

### 4.3. Association between Fall Prevention Self-Efficacy and Falls

Previous studies reported that low fall prevention self-efficacy or FOF in older adults was highly associated with the occurrence of falls [14,20,29]. In this study, low fall prevention self-efficacy was also associated with the occurrence of falls. On the other hand, regarding the distribution of MFES scores among non-fallers and fallers, the fall group showed a polarization between distributional tendencies toward low and high scores and included individuals who had high self-efficacy scores but had fallen. In fact, Sugihara et al. [16] noted that a certain number of individuals with a fall history overestimated their own abilities as self-efficacy. Logistic regression analysis using Bayesian estimation and comparison of ORs for the occurrence of falls for each factor revealed that ORs for each cognition-error item in the four physical performance assessments ranged from 3.1 to 170.72, whereas ORs for fall prevention self-efficacy ranged from 0.97 to 1.0. Therefore, fall prevention self-efficacy was less influential as a risk factor in predicting fall risk than cognition error in physical performance, and some aspects of fall prevention self-efficacy scores, as standalone factors, did not accurately assess fall risk.

### 4.4. Limitations

In the present cross-sectional study, we examined the association of each variable with falls by calculating the risk of occurrence of falls in the fall group when the variables of fall prevention self-efficacy and deviation in self-cognition of physical performance were used, based on differences in odds ratios, but the interrelationship between the two variables was not clear. In particular, in the distribution of MFES scores, the fall group showed a polarization between low and high trends and included those who fell even though their fall prevention self-efficacy scores were high, but the degree of influence of the difference in MFES scores in the two groups has not been fully examined. 

In this study, Bayesian statistics were used to analyze factors related to falls. Bayesian statistics is a method that allows the parameters and overall trend to be estimated as random variables using random sampling, even with a small number of samples, in contrast to conventional statistical methods that require a large number of samples to be collected and verified [35,36]. The parameter of interest in this study was self-cognition of physical performance in relation to the presence or absence of falls, and the posterior probability of this parameter was determined using the Markov chain Monte-Carlo method (MCMC) by generating random numbers from a multivariate probability distribution and extracting a sample. The posterior probability of each parameter was obtained by random sampling of a total of 4000 samples (2000 for iteration and four chains, and 1000 warm-up periods), for random number generation as is standard practice in Bayesian statistics.

In this study, the results of Bayesian statistics show that the R hat is less than 1.1 for all parameters, indicating that the posterior distribution of the parameter we want to find has converged, which is an acceptable method for statistical validation. However, it would be desirable to increase the number of subjects in the future in order to further divide them into groups according to differences in MFES scores and compare the impact of these differences by focusing only on the fall group. 

The deviation in self-cognition of physical performance was a more explanatory factor for the fall risk, and there was a tendency to overestimate the fall risk for all physical performance. However, the non-fall group also showed a tendency to overestimate their self-cognition of physical performance, which may lead to falls in the future. In order to clarify fall factors in more detail in the future, it is important to examine differences in the diseases of the older adults studied, as well as differences in age, a strong fall-related factor, and thus differences in the effects of age-related changes. It would also be desirable to use a longitudinal study as the research design to explore the association between the presence or absence of falls since the study was conducted.

### 4.5. Further Study

The occurrence of falls is thought to be influenced by the individual’s ability to adapt to an environment where internal and external factors interact. The suggestion from our study that a deviation in self-perception was highly associated with falls may support this idea. Therefore, in future consideration of fall prevention interventions for older adults, along with exercise interventions that have been recommended in the past, it is also important for the participants themselves to be aware of their own condition, which is an internal factor, and to appropriately identify fall-related factors in the environment, which is an external factor, and to increase their self-cognition of their physical performance. Devising and developing new methods to support older adults so that they can take appropriate actions in their daily lives will be an important issue in the future.

## 5. Conclusions

This study revealed that lower fall prevention self-efficacy and greater deviation in self-cognition of physical performance were highly associated with falls, regardless of the degree of physical function decline in the fall group as compared to the non-fall group. These results also suggested that deviation in self-cognition of physical performance was a factor with more explanatory power for the occurrence of falls. Furthermore, older adults, such as the participants in this study, who already had some disability and were living their daily lives with some support, tended to overestimate all physical performance. Among them, overestimation of timed cognitive ability, which is a better indicator of motor imagery ability than spatial cognitive ability, was significantly associated with the occurrence of falls.

## Figures and Tables

**Table 1:** Comparison of characteristics of the fall and non-fall groups.

	Fall Group (*n* = 14)		Non-Fall Group (*n* = 13)	
Age, mean (SD ^1^), years	79.64	(8.22)	83.31	(6.60)
Sex, *n* (%)				
Male	4	(28.6)	2	(15.4)
Female	10	(71.4)	11	(57.1)
Learning Experience, *n* (%)	12	(85.7)	8	(61.5)
Care requirement level, *n* (%)				
Support level 1	2	(14.3)	5	(38.5)
Support level 2	8	(57.1)	4	(30.8)
Long-term care level 1	1	(7.1)	4	(30.8)
Long-term care level 2	1	(7.1)	0	(0.0)
Long-term care level 3	0	(0.0)	0	(0.0)
Long-term care level 4	2	(14.3)	0	(0.0)
Long-term care level 5	0	(0.0)	0	(0.0)

^1^ SD = standard deviation.

**Table 2:** Comparison of test results between the fall and non-fall groups.

	Fall Group (*n* = 14)		Non-Fall Group (*n* = 13)	
	Mean	SD	Mean	SD
HDS-R ^1^	27.1	2.89	27.5	1.76
MFES ^2^	83.2	42.67	103.2	20.3
LSA ^3^	40.1	21.3	53.2	16.5
Grip (kg)	20.8	7.26	17.4	4.6
Estimated FRT ^4^ (cm)	23.11	8.83	27.15	10.46
Actual FRT (cm)	22.12	10.09	22.42	8.37
FRT cognition error *	1.42	1.25	1.26	0.5
Estimated stepping over test (cm)	32.61	9.15	31.26	8.77
Actual stepping over test (cm)	37.43	15.76	32.7	12.55
Stepping over test cognition error **	1.38	1.75	1.03	0.35
Estimated TUG ^5^ (normal) (s)	11.09	7.8	7.84	3.1
Actual TUG (normal) (s)	19.3	12.25	15.82	4.42
TUG (normal) cognition error ***	0.6	0.21	0.52	0.2
Estimated TUG (rapid) (s)	8.86	6.72	5.42	1.92
Actual TUG (rapid) (s)	15.81	9.18	12.64	4.44
TUG (rapid) cognition error ****	0.55	0.27	0.45	0.14

^1^ HDS-R = revised Hasegawa dementia scale; ^2^ MFES = modified fall efficacy scale; ^3^ LSA = life-space assessment; ^4^ FRT = functional reach test; ^5^ TUG = timed up and go test. * FRT cognition error = estimated FRT/actual FRT. ** Stepping over test cognition error = estimated stepping over test/actual stepping over test. *** TUG (normal) cognition error = estimated TUG (normal)/actual TUG (normal). **** TUG (rapid) cognition error = estimated TUG (rapid)/actual TUG (rapid).

**Table 3:** Correlation with fall history for each factor.

	Correlation Coefficient with Fall History, r	*p*-Value
Sex ^†^		0.648
Learning Experience ^†^		0.209
Care requirement level ^††^	0.328	0.069
Age ^†††^	−0.246	<0.001
MFES ^1 †††^	−0.294	<0.001
LSA ^2 †††^	−0.335	<0.001
Grip ^†††^	0.276	<0.001
FRT ^3^ cognition error ^†††^	0.088	<0.001
Stepping over test cognition error ^†††^	0.141	<0.001
TUG ^4^ (normal) cognition error ^†††^	0.199	<0.001
TUG ^4^ (rapid) cognition error ^†††^	0.232	<0.001

^1^ MFES = modified fall efficacy scale; ^2^ LSA = life-space assessment; ^3^ FRT = functional reach test; ^4^ TUG = timed up and go test. ^†^ Exact probability test; ^††^ Polychoric correlation; ^†††^ Point-sequence correlation.

**Table 4:** Association between fall history and each factor by logistic regression analysis with each cognition error added as a variable (**a**–**d**).

**(a) FRT**						
	**Estimate**	**Estimate Error**	**CI** **Lower 95%**	**CI** **Upper 95%**	**Rhat**	**Odds Ratio**
Age	−0.23	0.12	−0.49	−0.02	1	0.79
MFES ^1^	−0.01	0.02	−0.05	0.03	1	0.99
LSA ^2^	−0.11	0.05	−0.23	−0.02	1	0.9
Grip	0.32	0.14	0.09	0.62	1	1.38
FRT ^3^ cognition error	1.13	0.86	−0.35	3.05	1	3.1
**(b) Stepping over test**						
	**Estimate**	**Estimate Error**	**CI** **Lower 95%**	**CI** **Upper 95%**	**Rhat**	**Odds Ratio**
Age	−0.26	0.13	−0.55	−0.04	1	0.77
MFES ^1^	0	0.03	−0.04	0.06	1	1
LSA ^2^	−0.14	0.06	−0.28	−0.03	1	0.87
Grip	0.33	0.13	0.1	0.62	1	1.39
Stepping over test cognition error	2.25	1.45	−0.01	5.63	1	9.49
**(c) TUG (normal speed)**						
	**Estimate**	**Estimate Error**	**CI** **Lower 95%**	**CI** **Upper 95%**	**Rhat**	**Odds Ratio**
Age	−0.18	0.12	−0.44	0.03	1	0.83
MFES ^1^	−0.03	0.02	−0.08	0.02	1	0.97
LSA ^2^	−0.11	0.05	−0.23	−0.02	1	0.9
Grip	0.23	0.12	0.02	0.51	1	1.26
TUG ^4^ (normal) cognition error	5.14	3.62	−1.23	13.07	1	170.72
**(d) TUG (maximum rapid speed)**						
	**Estimate**	**Estimate Error**	**CI** **Lower 95%**	**CI** **Upper 95%**	**Rhat**	**Odds Ratio**
Age	−0.2	0.12	−0.47	0.02	1	0.82
MFES ^1^	−0.02	0.02	−0.07	0.02	1	0.98
LSA ^2^	−0.12	0.06	−0.25	−0.02	1	0.89
Grip	0.22	0.12	0.01	0.49	1	1.25
TUG ^4^ (rapid) cognition error	5.13	3.58	−1.23	12.62	1	169.02

^1^ MFES = modified fall efficacy scale; ^2^ LSA = life-space assessment; ^3^ FRT = functional reach test; ^4^ TUG = timed up and go test.

## Data Availability

Not applicable.
